# Divergent evolution in bilateral prostate cancer: a case study

**DOI:** 10.3389/fonc.2025.1681073

**Published:** 2026-09-10

**Authors:** Roni Haas, Rong Rong Huang, Adam B. Weiner, Yash Patel, Lydia Y. Liu, Takafumi N. Yamaguchi, Raag Agrawal, Paul C. Boutros, Robert E. Reiter

**Affiliations:** 1 Department of Human Genetics, University of California Los Angeles, Los Angeles, CA, United States; 2 Department of Urology, University of California Los Angeles, Los Angeles, CA, United States; 3 Jonsson Comprehensive Cancer Center, University of California Los Angeles, Los Angeles, CA, United States; 4 Institute for Precision Health, University of California Los Angeles, Los Angeles, CA, United States; 5 Department of Pathology, University of California Los Angeles, Los Angeles, CA, United States

**Keywords:** clonal disease, driver CNA regions, multifocal prostate cancer, precision oncology, WGS characterization

## Abstract

Multifocal prostate cancer is a prevalent phenomenon, with most cases remaining uncharacterized from a genomic perspective. A patient presented with bilateral prostate cancer. On systematic biopsy, two indistinguishable clinicopathologic lesions were detected. Whole-genome sequencing displayed somatically unrelated tumors with distinct driver CNA regions, suggesting independent origins of the two tumors. We demonstrated that similar clinicopathologic multifocal tumors, which might be interpreted as clonal disease, can in fact represent independent cancers. Genetic prognostics can prevent mischaracterization of multifocal disease to enable optimal patient management.

## Highlight

**Figure 1:**
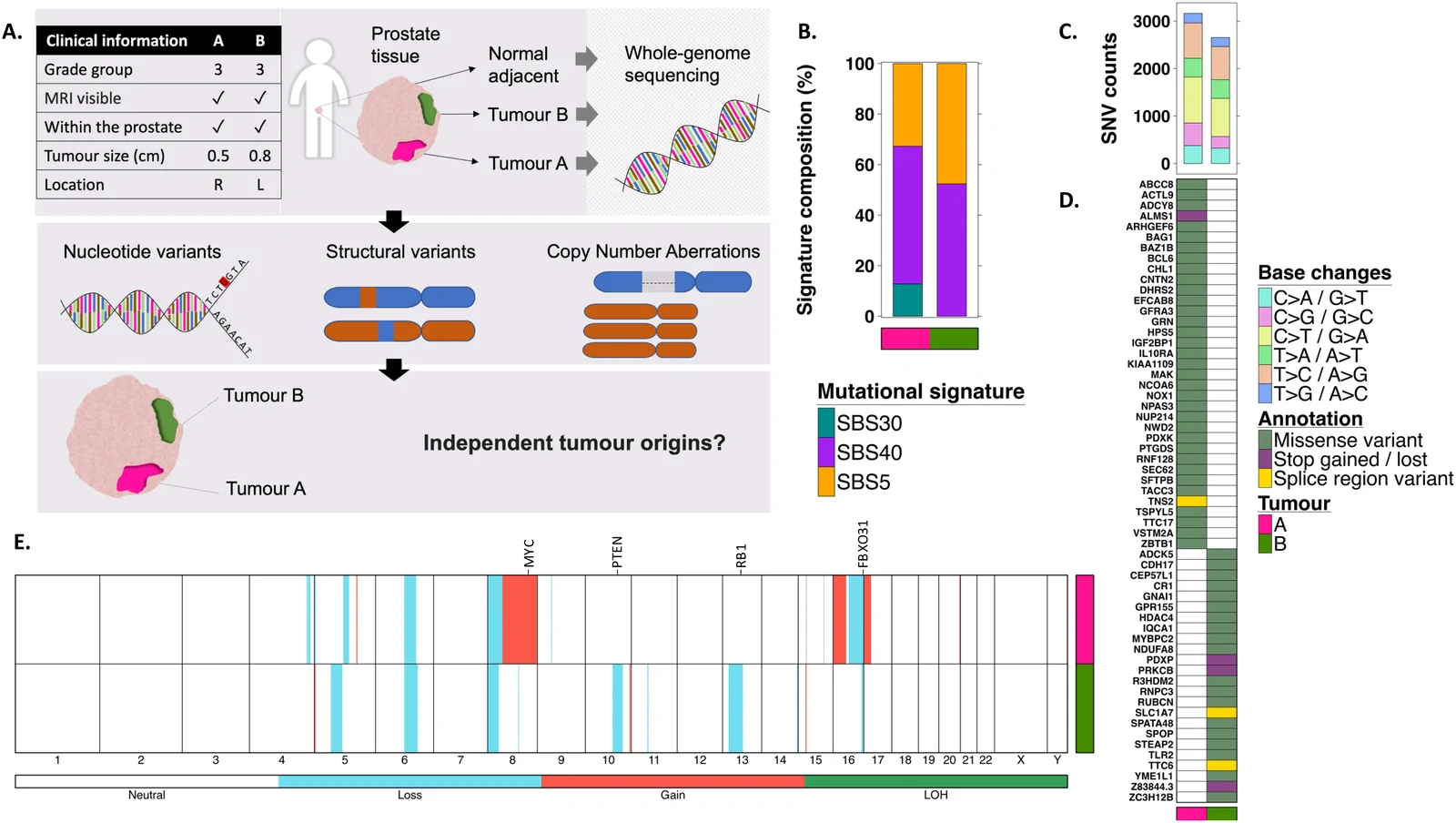
Genomic landscape. **(A)** Schematic of study design and workflow. **(B)** Mutational signatures. Signature etiologies: SBS30, defective DNA base excision repair due to NTHL1 mutation; SBS40, unknown; SBS5, unknown. **(C)** SNV counts categorized by the base change type. **(D)** Functionally affected genes by SNVs **(E)** Genome-wide view of CNAs. Chromosome numbers are presented on the x-axis. The colored lines represent CNA classes. LOH, loss of heterozygosity.

In this report, we investigated the bilateral prostate cancer of a patient in his 60s, who is genetically similar to the European reference population (**Supplementary Table S1**). The patient was diagnosed with bilateral Gleason Grade (GG) 1 tumors on systematic biopsy and was upgraded to GG3 in both on pathology from surgical specimens. Both tumors harbored 60% pattern 4 and 4% tertiary pattern 5. There were no cribriform or intraductal components and both tumors were confined to the prostate. The uncanny clinical and histologic similarities between the two tumors (**Figure 1A**) inspired molecular evaluations to test if nearly indistinguishable clinicopathologic features reflect similar tumor biology.

We sampled the separate tumors (A and B) from freshly frozen radical prostatectomy tissue. Histologically normal (benign) prostate tissue, procured simultaneously from the same patient, was used as a reference control. DNA whole genome sequencing (WGS) was carried out to interrogate the genomic landscape and evolutionary relationship of the tumors (**Figure 1A**; **Supplementary Figure S1**).

We first evaluated the extent of genomic instability in each tumor as reflected by mutation counts. Larger numbers of somatic single nucleotide variants (SNVs) and structural variants (SV) were detected in tumor A compared to B, and none were shared between the two (**Table 1**; **Supplementary Tables S2, S3**; **Supplementary Figure S2**). The SNVs in both tumors were mostly the consequences of similar mutagenic processes, although the SBS30 signature was only found in Tumor A (**Figure 1B**). The tumors experienced similar profiles of base change mutations (**Figure 1C**) and contained multiple SNVs that are predicted to have a significant protein functional effect. No genes shared functional SNV in both tumors (**Figure 1D**). Among driver genes, *BCL6* and *NUP214* were functionally affected in Tumor A, and *SPOP* in Tumor B.

**Table 1:** Mutation burden.

	Tumor A	Tumor B	Shared
SNV	3,164	2,654	0
SV	55	41	0
Gains	7	3	0
Loses	8	10	3
LOH	1	0	0

^1^Somatic single nucleotide variant (SNV), somatic structural variants (SV), loss of heterozygosity (LOH).

Next, we compared the copy number aberration (CNA) profiles of tumors A and B to identify possible shared CNA-driver regions, which could imply a shared evolutionary origin (**Table 1**; **Figure 1E**; **Supplementary Table S4**). Three CNA regions overlapped (**Supplementary Figure S3**), but only one, on chromosome 16, was a CNA-driver region spanning *FBXO3* (**Supplementary Figure S3C**). However, the breakpoints of these events differ, indicating convergent evolution rather than a shared origin.

Consistent with SNV and SV results, slightly higher genomic instability originating from CNAs was observed in Tumor A (**Table 1**). This involved CNA-driver regions, including *MYC* and *NCOA2* gains on chromosome 8q and *NKX3–1* deletion on chromosome 8p, typically seen in prostate cancer. By contrast, Tumor B was characterized by *PTEN* deletion on chromosome 10, and *RB1* deletion on chromosome 13. Overall, the CNA landscape displayed distinctive clonal drivers.

Immunohistochemical staining confirmed major differences between the tumors, including in AMACR, PTEN and PSMA. Similarly, *PTEN* deletion in tumor B only was also corroborated by immunohistochemistry (**Supplementary Figure S4**).

Oncotype DX Genomic Prostate Score (GPS) (1, 2) for Tumor B indicated a favorable intermediate risk, suggesting that tests like GPS should be conducted on both regions in cases of bilateral prostate cancer.

Taken together, our results support a distinct genetic origin of the two tumors, indicating that they arose independently and represent two separate cancers. The remarkably similar clinicopathologic features could have been mistakenly considered clonal without further genetic characterization. Our work represents one of the few WGS-based prostate cancer multifocal case studies demonstrating genetic independence of morphologically similar foci (3–5). This case, and previous works (3–9), emphasize that mischaracterizing multifocal prostate tumors, which are relatively common (10, 11), as a single prostate cancer may prevent optimal patient management. Such genomic distinctions can inform precision oncology, where identifying independent tumor foci could help guide tailored patient management. Our study encourages genetic characterization for a deeper understanding of multifocal Tumor biology and ultimately shaping future clinical practices.

## Methods

### Sample preparation and WGS

Fresh prostate tissue samples were procured from a patient who underwent radical prostatectomy. Eight tissue specimens were grossed from four quadrants at prostate levels 2 and 4 and immediately embedded in Optimal Cutting Temperature (OCT) compound. Frozen sections were cut from all eight OCT blocks for hematoxylin and eosin (H&E) staining. A research pathologist conducted detailed pathological annotations, including tumor content, cellularity, Gleason score (GS), intraductal carcinoma (IDC), large cribriform pattern, ductal differentiation, comedonecrosis, and tumor-infiltrating lymphocytes (TILs), which were subsequently verified by a board-certified genitourinary pathologist.

Two representative tumor blocks were selected for molecular analysis: one from level 2 anterior right (2AR) with GS 3 + 3 = 6, designated as Tumor A, and another from level 4 posterior left (4PL) with GS 4 + 3 = 7, designated as Tumor B. A true tumor-free distal normal prostate block from level 2 anterior left (2AL) was selected as the matched normal reference. Regions of interest (ROIs) with greater than 80% tumor cellularity were macro-dissected from Tumor A and Tumor B for nucleic acid isolation. Prior to macro-dissection, ten unstained 3-μm sections were cut from each tumor block and mounted on the same slide to ensure that post-validation immunohistochemistry (IHC) staining was performed under identical conditions, allowing for side-by-side comparison with minimal variability.

Tumor DNA was extracted from the macro-dissected ROIs using the Qiagen AllPrep DNA Mini Kit (Qiagen, Cat. 80204). For consistency, the tissue used for DNA extraction was obtained from the same ROIs within the same OCT blocks utilized for IHC for both tumors. Normal DNA was isolated from the normal prostate tissue using the Qiagen QIAamp DNA Mini Kit (Qiagen, Cat. 51304). Libraries were constructed using 500–1000 ng of DNA with the KAPA HyperPrep Kit (Roche, Cat. KR0961). Whole genome sequencing was performed on the Illumina NovaSeq 6000 S4 platform using 150-bp paired-end reads across 1.5 lanes, achieving approximately 120× coverage for tumor samples and 80× coverage for normal prostate tissue. Data quality was assessed using Illumina Sequencing Analysis Viewer (SAV), and demultiplexing was performed using Illumina Bcl2fastq v2.19.1.403.

### WGS processing and mutation calling

The process was based on the Metapipeline-DNA pipeline (12). DNA reads were tested with FastQC v0.11.8 (13) for quality assurance. The reads were mapped to the human GRCh38 reference genome using BWA-MEM2 v2.2 (14). Aligned SAM files were converted to BAM files using SAMtools v1.12 (15). Using Picard Tool’s v2.26.10 (16), the BAM files were sorted in coordinate order, duplicates were marked, and the BAM files were indexed. Indexed BAM files went through indel-realignment using the Genome Analysis Toolkit (GATK) v3.7.0 (17). Base quality score recalibration was done using GATK v4.1.9.0 (17) in tumor-normal pairs. Separate BAM files were generated for the tumor and normal samples, and their headers were modified using SAMtools v1.12 (15). Qualimap v.2.2.2-dev (18) was used to verify high coverage (**Supplementary Figure S1**). The mean coverage was 105X for T1, and 95X for T2. We verified that all samples originated from the same patient by comparing the germline genotypes of each tumor with the matched normal sample, observing over 99.9% concordance for both tumors.

Processed BAM files were taken for mutation calling in tumor-normal pairs. SNVs were called separately using, Mutect2 of GATK v4.2.0.0 (17), Strelka2 v2.9.10 (19) and SomaticSniper v1.0.5.0 (20). SNVs were declared only for sites that were detected using at least two algorithms for each tumor site. Germline variant filtering was performed, excluding any variant in the normal control sample and in gnomAD v3.1.2 (21). SNVs were annotated using SnpEff v5.0e (22). Mutational signatures were identified using SigProfilerExtractor v1.14 (23).

SVs were identified using DELLY v0.8.7 (24). Detected sites were filtered to exclude germline structural variants. SVs that were identified with DELLY, despite very low coverage in their area in the normal sample, were excluded. Two deletions in Tumor A that were larger than 25Mb with no CNA evidence in the corresponding regions were excluded due to low confidence. CNAs were detected using Battenberg v2.2.9 (25), with a purity of 0.41 and 0.21 for tumors A and B respectively, and ploidy of ~2 for both. Each call was reviewed manually to filter out false findings.

### Immunohistochemistry assay

Frozen slides from Tumor A and Tumor B were retrieved from –80°C, air-dried, and fixed in 10% cold neutral-buffered formalin. IHC was performed on the Agilent AutoStainer Link 48 using customized protocols for single, dual, triple, and quadruple chromogen-based assays. Background was reduced using EnVision Flex Peroxidase Block (Agilent, Cat. K8002) and casein blocking solution (Vector Laboratories, Cat. SP-5020).

Triple and quadruple IHC panels included primary antibodies targeting p63 (BioCare, CM163), high-molecular-weight cytokeratin (Agilent, M0630), CD3 (Agilent, A0452), CD8 (Agilent, M7103), CD4 (Cell Marque, 104R), AMACR (Agilent, M3616), PSMA (Agilent, M3620), PSCA (Santa Cruz, SC-80654), and PIGR (Millipore Sigma, HPA012012). Dual IHC was performed using ERG (Abcam, ab92531) and SPINK1 (Abcam, ab183034), while single IHC assays employed PTEN (Agilent, M3627) and RB (Santa Cruz, SC-102). IHC conditions are described in **Supplementary Table S5**.

Signal visualization was performed using Leica post-primary reagent (RE7150-CE) and Novolink polymer (RE7140-CE) in combination with Flex DAB+, Magenta, Vector HRP Violet, AP Blue, and BioCare HRP Green substrate-chromogens. Counterstaining was carried out using EnVision Flex Hematoxylin. Negative controls included slides with omitted primary antibodies as well as Flex negative control mouse cocktail (IgG1, IgG2a, IgG2b, IgG3, IgM) and/or non-immunized rabbit serum.

### Genetic ancestry

Genetic ancestry was predicted using Peddy v0.4.8, with WGS of the normal sample as input (26) (**Supplementary Table S1**).

### Visualization

Visualizations were generated in the R environment v4.2.0 using the BoutrosLab.plotting.general package v7.0.3 (27).

## Data Availability

The original contributions presented in the study are publicly available. This data can be found here: The associated dbGaP; accession number phs004832; https://www.ncbi.nlm.nih.gov/projects/gap/cgi-bin/study.cgi?study_id=phs004832.v1.p1.
