# Supplementary material for: Divergent evolution in bilateral prostate cancer: a case study
Source: Front Oncol. 2026 Sep 10;15:1681073. doi: 10.3389/fonc.2025.1681073 (PMC13601985; doi:10.3389/fonc.2025.1681073)
Supplement: Supplementary file 1 [file DataSheet1.pdf]

Supplementary Figures

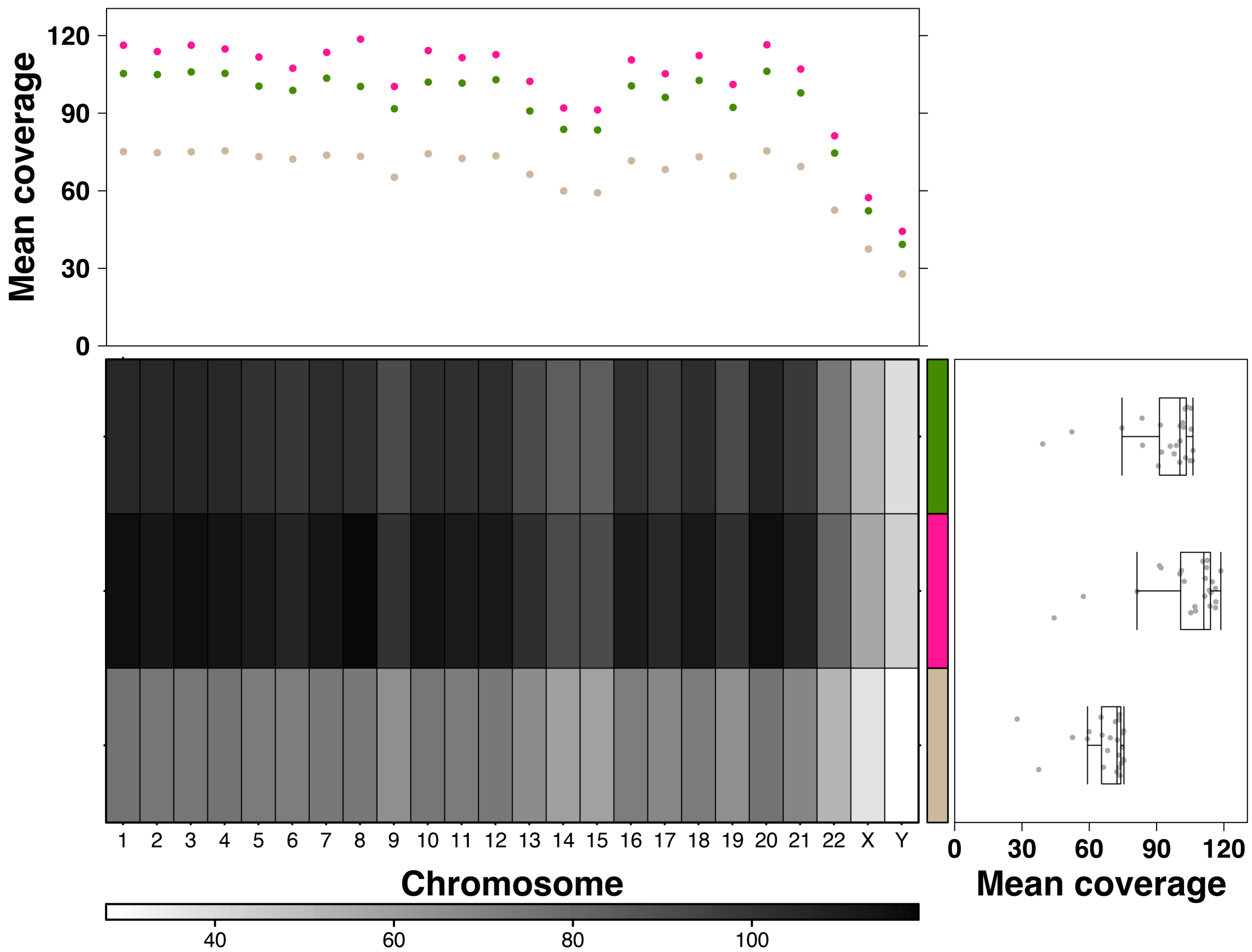

Supplementary Figure 1. Read coverage across the genome. Chromosome numbers are presented on the x-axis. The heatmap colors show the read coverage. Pink, tumour A; Green, tumour B, Tan, normal sample.

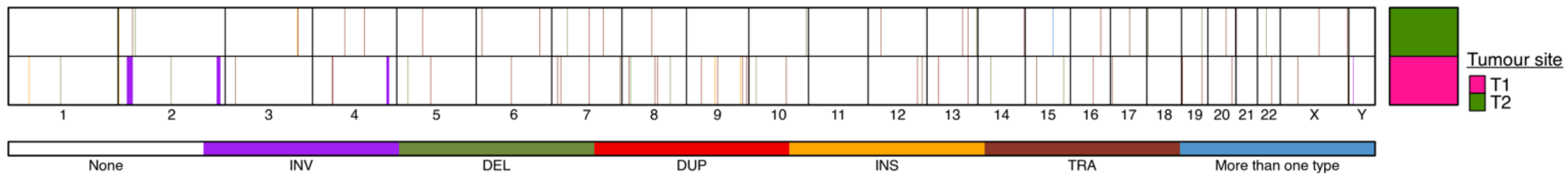

Supplementary Figure 2. The SV profiles of Tumours A and B. Chromosome numbers are presented on the x-axis. The colored lines represent types of SVs: INV, inversion; DEL, deletion; DUP, duplication; INS, insertion; TRA, translocation. Pink, Tumour A, Green Tumour B.

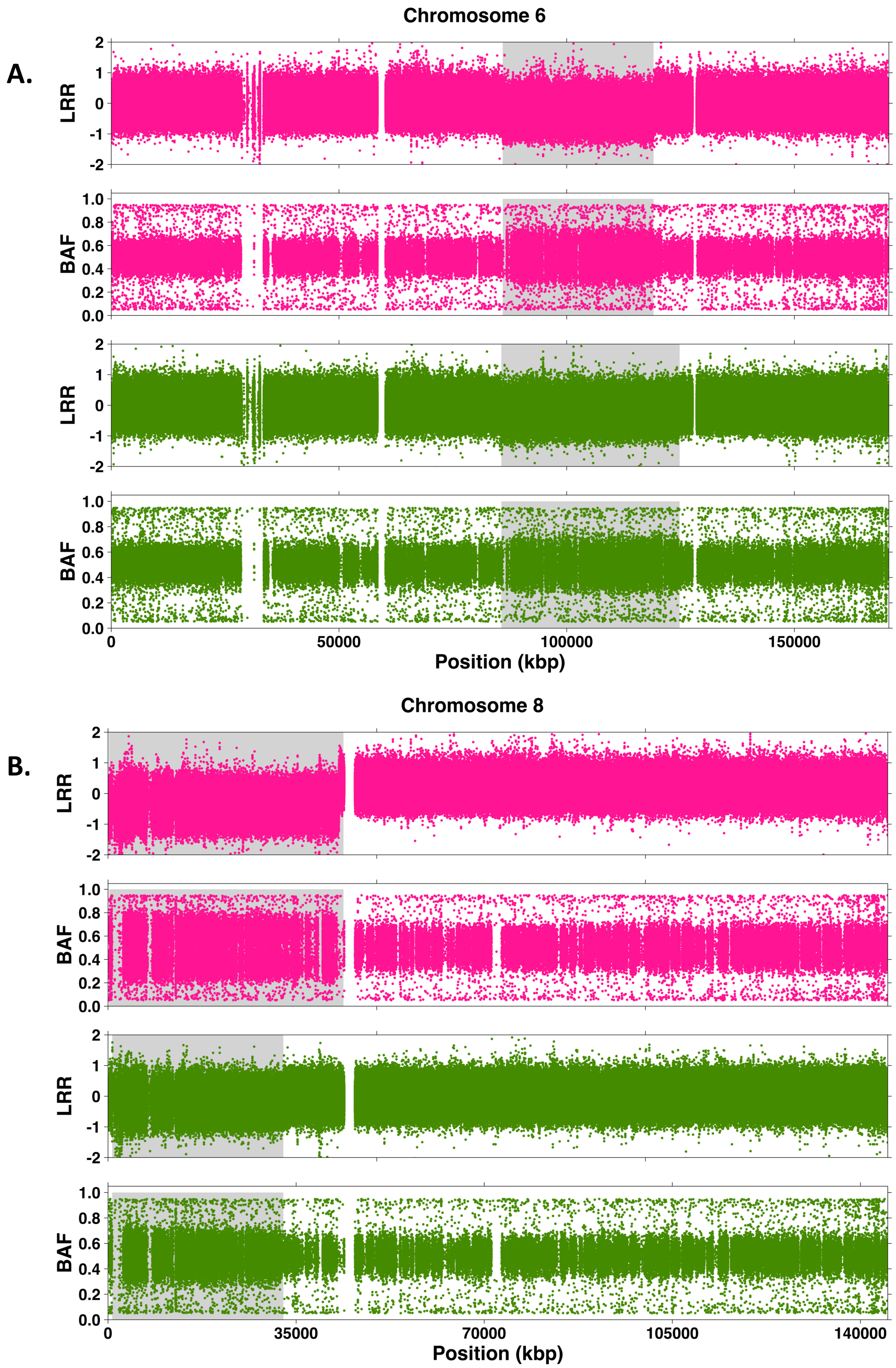

Supplementary Figure 3. Shared CNA regions. Presented are CNA regions that show similar log R ratio (LRR) and B- Allele frequencies (BAF) patterns between Tumour A and B in chromosomes 6 (**A**), 8 (**B**) and 16 (**C**). Homozygous SNP (BAF > 0.95 or BAF < 0.05) were excluded for visualization. Pink, Tumour A; Green, Tumour B. Marked in grey are the CNA regions.

C.

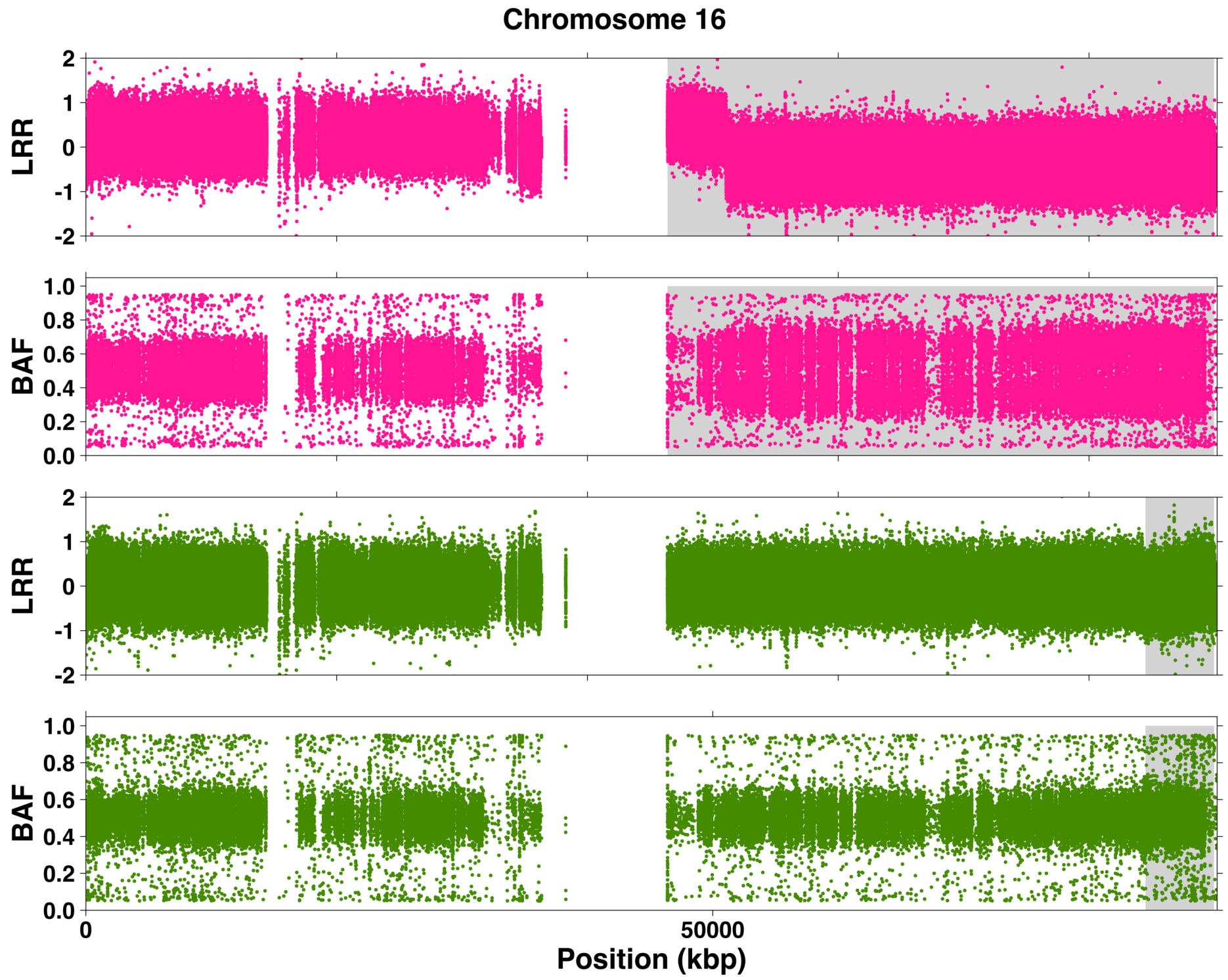

Supplementary Figure 3. Shared CNA regions. Presented are CNA regions that show similar log R ratio (LRR) and B- Allele frequencies (BAF) patterns between Tumour A and B in chromosomes 6 (**A**), 8 (**B**) and 16 (**C**). Homozygous SNP (BAF > 0.95 or BAF < 0.05) were excluded for visualization. Pink, Tumour A; Green, Tumour B. Marked in grey are the CNA regions.

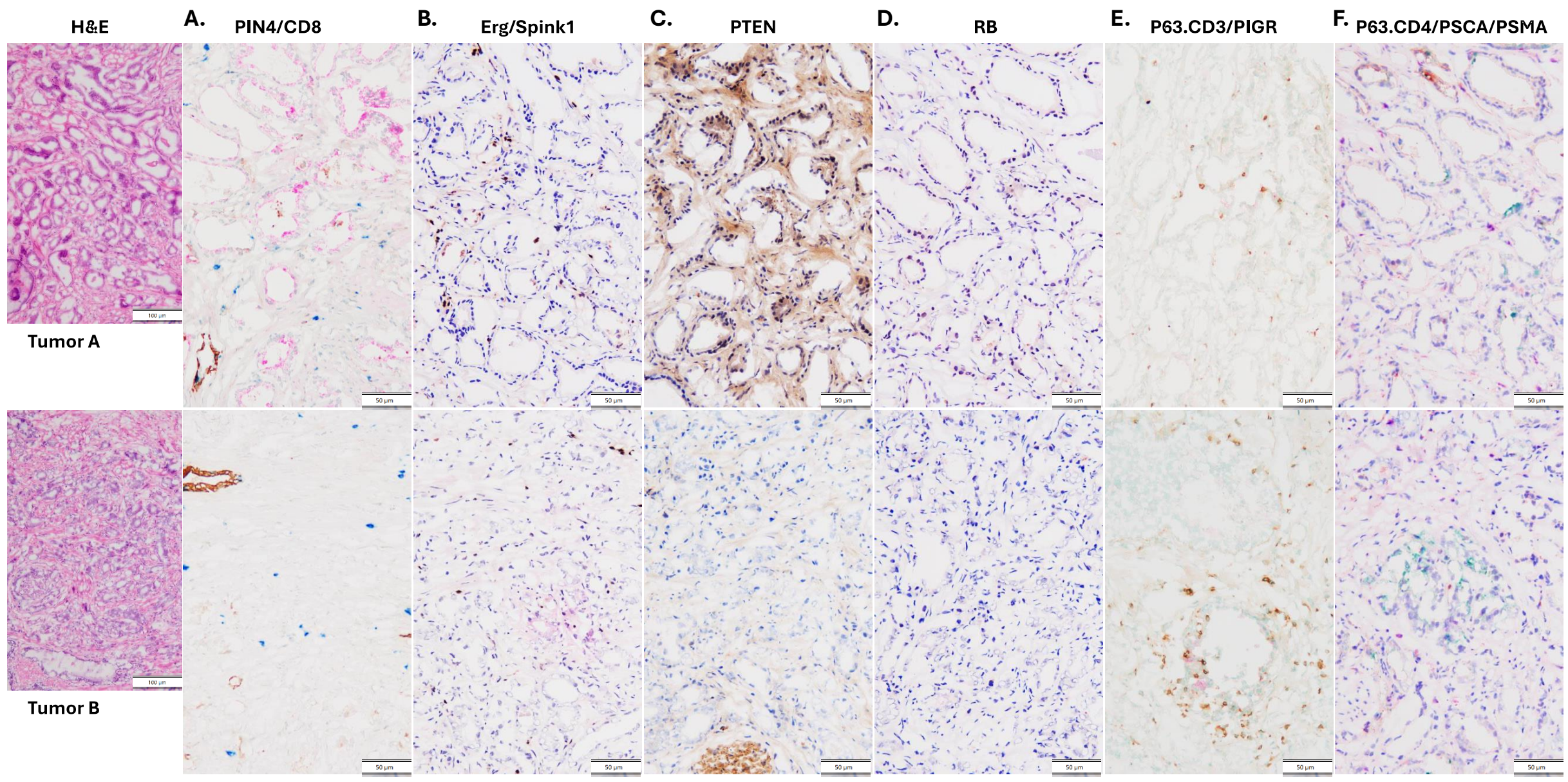

Supplementary Figure 4. Immunohistochemical staining reveals differences between the two tumours. A. PIN4 staining shows higher AMACR (red) protein abundance in the lumens of Tumour A compared to B, with minimal CD8+ cytotoxic T-cell infiltration in both tumours. The higher abundance of AMACR in Tumour A compared to B was assessed semi-quantitatively, based on both staining intensity and the percentage of positive Tumour cells. **B.** Neither Tumour A nor B expresses ERG (brown), and a small cluster of cells in Tumour B is positive for Spimk1/12 (red). **C.** PTEN protein (brown) is retained in Tumour A and is lost in Tumour B, even in a few adjacent normal glands (not shown). **D.** Minimal differences in RB abundance (brown) are observed between Tumour A and B. **E.** A few Tumour cells express PIGR (red) attracting more intratumorally CD3+ Pan T cells (brown) infiltration in Tumour B compared to A. **F.** PSMA (green) abundance is higher in Tumour B compared to A, while PSCA (brown) could not be detected in both. Few intratumorally CD4+ Helper T cells (red) are observed in both tumours.
